# Induced Pluripotent Stem Cell‐Derived Endothelial Cells Overexpressing Interleukin‐8 Receptors A/B and/or C‐C Chemokine Receptors 2/5 Inhibit Vascular Injury Response

**DOI:** 10.1002/sctm.16-0316

**Published:** 2017-02-24

**Authors:** Samantha Giordano, Xiangmin Zhao, Yiu‐Fai Chen, Silvio H. Litovsky, Fadi G. Hage, Tim M. Townes, Chiao‐Wang Sun, Li‐Chen Wu, Suzanne Oparil, Dongqi Xing

**Affiliations:** ^1^ Vascular Biology and Hypertension ProgramDivision of Cardiovascular Disease, Department of Medicine, University of Alabama at BirminghamBirminghamAlabamaUSA; ^2^ Division of Anatomic PathologyDepartment of Pathology, University of Alabama at BirminghamBirminghamAlabamaUSA; ^3^ Section of Cardiology, Birmingham Veterans Affairs Medical CenterBirminghamAlabamaUSA; ^4^ Department of Biochemistry and Molecular GeneticsUniversity of Alabama at BirminghamBirminghamAlabamaUSA

**Keywords:** iPS‐endothelial cells, Targeted cell therapy, Vascular injury, Interleukin 8 receptor, C‐C chemokine receptor 2/5

## Abstract

Recruitment of neutrophils and monocytes/macrophages to the site of vascular injury is mediated by binding of chemoattractants to interleukin (IL) 8 receptors RA and RB (IL8RA/B) C‐C chemokine receptors (CCR) 2 and 5 expressed on neutrophil and monocyte/macrophage membranes. Endothelial cells (ECs) derived from rat‐induced pluripotent stem cells (RiPS) were transduced with adenovirus containing cDNA of IL8RA/B and/or CCR2/5. We hypothesized that RiPS‐ECs overexpressing IL8RA/B (RiPS‐IL8RA/B‐ECs), CCR2/5 (RiPS‐CCR2/5‐ECs), or both receptors (RiPS‐IL8RA/B+CCR2/5‐ECs) will inhibit inflammatory responses and neointima formation in balloon‐injured rat carotid artery. Twelve‐week‐old male Sprague‐Dawley rats underwent balloon injury of the right carotid artery and intravenous infusion of (a) saline vehicle, (b) control RiPS‐Null‐ECs (ECs transduced with empty virus), (c) RiPS‐IL8RA/B‐ECs, (d) RiPS‐CCR2/5‐ECs, or (e) RiPS‐IL8RA/B+CCR2/5‐ECs. Inflammatory mediator expression and leukocyte infiltration were measured in injured and uninjured arteries at 24 hours postinjury by enzyme‐linked immunosorbent assay (ELISA) and immunohistochemistry, respectively. Neointima formation was assessed at 14 days postinjury. RiPS‐ECs expressing the IL8RA/B or CCR2/5 homing device targeted the injured arteries and decreased injury‐induced inflammatory cytokine expression, neutrophil/macrophage infiltration, and neointima formation. Transfused RiPS‐ECs overexpressing IL8RA/B and/or CCR2/5 prevented inflammatory responses and neointima formation after vascular injury. Targeted delivery of iPS‐ECs with a homing device to inflammatory mediators in injured arteries provides a novel strategy for the treatment of cardiovascular diseases. Stem Cells Translational Medicine *2017;6:1168–1177*

**Significance Statement**

One major unsolved problem for cell‐based therapy is how to home transplanted cells to damaged organs to improve their survival and to enhance tissue repair and organ function. When equipped with certain chemokine receptors as a homing device, induced pluripotent stem cell‐derived endothelial cells mimic the behavior of neutrophils/monocytes and compete with the binding of these cells to chemokines overexpressed in injured tissue, thus inhibiting inflammatory response and adverse vascular remodeling following vascular injury. The results of the present study open the door to new therapeutic applications of cell therapy in patients with cardiovascular injury.

## Introduction

Vascular inflammation that results from endothelial injury is a major contributor to most forms of cardiovascular disease (CVD). We have shown that the response to acute endoluminal vascular injury occurs in two phases 1, 2, 3, 4, 5, 6, 7. The first phase, which begins immediately after injury and lasts for ∼48 hours, is characterized by increased inflammatory cytokine production and leukocyte (neutrophil, monocyte/macrophage, and T‐cell) infiltration into the injured vasculature 1, 4, 8, 9, 10. Previous work in the rat carotid artery balloon injury model demonstrated a significant increase in expression of cytokine‐induced neutrophil chemoattractant (CINC)−2β (human homolog of IL8) and C‐C chemokine ligand 2 (CCL2)/monocyte chemoattractant protein‐1 (MCP‐1) in injured arteries within 24 hours postinjury 1, 4, 8, 9, 10. The second phase, which lasts for ∼4 weeks in rodents, is characterized by an increase in fibroblast and smooth muscle cell proliferation, myofibroblast transformation, and migration toward the lumen to form the neointima 3, 5, 6, 7, 10, 11.

Neutrophils expressing IL8A and B receptors are among the first wave responders that infiltrate injured tissues in response to locally expressed IL8 and exacerbate the inflammatory response by upregulating other inflammatory cytokines (e.g., CCL2/MCP‐1), causing influx of second wave responders, including monocytes/macrophages. We have shown that targeted cell therapy with adult rat aortic endothelial cells (RAECs) transfected with IL8 receptors A and B (IL8RA/B‐ECs) attenuates inflammation and neointima formation in balloon injured rat carotid arteries 10, 12.

Monocytes/macrophages express C‐C chemokine receptor type 2 (CCR2) and 5 (CCR5) receptors and migrate toward the monocyte/macrophage chemoattractants including CCL2/MCP‐1 that is expressed in injured tissues 13, 14. We have shown that CCL2/MCP‐1 mRNA and protein expression are increased in rat carotid arteries 24 hours after balloon injury and that monocytes/macrophages infiltrate injured arteries with 24 hours after vascular injury 1, 4, 10, 12, 15. Both neutrophils and monocytes/macrophages may contribute to inflammation‐stimulated neointima formation in injured arteries. In the current study, we used endothelial cells (ECs) overexpressing IL8RA/RB, CCR2/5, or both types of receptors as targeted cell therapy to define the roles of neutrophils and/or macrophages in the inflammatory response and neointima formation in balloon‐injured rat carotid arteries.

In the current study, we have extended our targeted cell delivery strategy to use of induced‐pluripotent endothelial stem cells (iPS‐ECs). We have prepared rat iPS (RiPS) cells by reprogramming fibroblasts isolated from rat embryos. The RiPS cells were oriented to differentiate into ECs and transduced with IL8RA/B and/or CCR2/5 for targeted cell therapy in rats with balloon‐induced carotid injuries. We hypothesized that RiPS‐ECs that overexpress IL8RA/B (PiPS‐IL8RA/RB‐ECs) and/or CCR2/5 (RiPS‐CCR2/5‐ECs) mimic the behavior of neutrophils and monocytes/macrophages that target and adhere to injured tissues, inhibit neutrophil and/or monocyte/macrophage infiltration, and attenuate inflammatory responses and structural and functional damage to injured arteries, thus enhancing vascular repair. We postulate that autologous iPS‐ECs may obviate immunologic concerns in future translational and clinical studies.

## Materials and Methods

### Cell Lines and In Vitro Studies

#### Reprogramming of Rat Fibroblasts into RiPS Cells

Rat embryonic fibroblasts (from E14.5 embryos) were isolated following the protocol described previously with slight modification 16, 17, 18, 19, 20. Isolated embryonic fibroblasts were de‐differentiated into iPS cells by transduction with polycistronic lentiviral vectors containing the genes for the transcription factors Oct4, Sox2, and Klf4, as described previously 16, 21. Cultured mitomycin‐inactivated embryonic fibroblasts (passage 3) were used as feeder cells in the iPS culture medium. The iPS culture medium was DMEM containing the supplements: 1x l‐glutamine, 1x penicillin/streptomycin, and 1x nonessential amino acids, 1x nucleosides, 15% FBS, 2‐mercaptoethanol, and leukemia inhibitory factor. An additional combination of MEK inhibitor (0.5 µM PD0325901), GSK3 inhibitor (3 µM CHIR99021), and ALK5 inhibitor (0.5 µM A‐83‐01) were added to the culture medium.

#### Characterization of RiPS Cells

RiPS cell colonies were cultured and expanded in iPS culture medium (with mitomycin‐inactivated rat embryonic fibroblast as feeder cells) for characterization. To evaluate the pluripotency of RiPS cells, cells were stained using the Vector Blue Alkaline Phosphatase Substrate Kit III (Vector Laboratories, Burlingame, CA, www.vectorlabs.com) according to the manufacturer's instructions 16, 21. RT‐PCR analysis was used as an additional method to confirm that the iPS cells expressed undifferentiated embryonic stem cell‐marker genes *Oct4* [16, 21]. In vivo teratoma formation was assessed to confirm pluripotency of RiPS cells 16. In brief, 1 × 10^6^ RiPS cells in 100 μl of phosphate‐buffered saline (PBS) were injected via a 21‐G needle into the dorsal flanks of severe combined immunodeficiency (SCID) mice. Teratomas were recovered 4–5 weeks postinjection and processed for histological analysis.

#### Differentiation and Characterization of RiPS‐ECs

Confluent cultures of RiPS cells were transferred to ultra‐low attachment dishes containing differentiation media for 4 days to form embryoid bodies (EBs). The differentiation media consisted of α‐Minimum Eagle's Medium, FBS (20%), β‐mercaptoethanol (0.05 mmol/l), nonessential amino acids (1%), bone morphogenetic protein‐4 (BMP‐4; 50 ng/ml), and vascular endothelial growth factor‐A (VEGF‐A, 100 ng/ml) (both from Peprotech, Rocky Hill, NJ). The 4‐day EBs were then seeded on 0.2% gelatin‐coated dishes and cultured for another 10 days in differentiation media. To purify the RiPS‐ECs, single cell suspensions were incubated with PE‐conjugated anti‐rat CD31 antibody. Flow cytometry was then performed to obtain purified RiPS‐ECs. The purified RiPS‐ECs were expanded in EC growth medium (MCDB‐131, VEC Technologies, Rensselaer, NY). The iPS‐ECs phenotype was confirmed by immunostaining with Abs against endothelial markers including endothelial nitric oxide synthase (eNOS), von Willebrand factor (vWF), and CD31 as described previously 12.

#### Characterization of Rat iPS‐ECs Transduced with IL8RA/B, CCR2/5, or AdNull Adenoviral Vectors

Rat iPS‐ECs overexpressing IL8RA/B, CCR2/5, or both receptors were generated using adenoviral vectors containing *IL8RA, IL8RB, CCR2, or CCR5 and green fluorescent protein (GFP)* genes using the AdEasy Adenoviral Vector System (Stratagene) as previously described 10, 12, 22, 23. RiPS‐ECs were grown to 80% confluence in EC growth medium (cat. no. MCDB‐131C, Vec Technologies, Rensselaer, NY) and treated with a mixture of adenoviruses containing *IL8RA‐GFP* and *IL8RB‐GFP*, *CCR2‐GFP* and *CCR5‐GFP*, or adenovirus containing only *GFP (AdNull‐GFP)* as a control. Eighteen hours after adenoviral transduction, fresh media was added to the cells, and 48 hours after transduction fluorescence imaging of the transduced ECs exhibited green fluorescence, confirming GFP expression 10, 12, 22, 23.

To confirm the expression of IL8RA/B and CCR2/5 in transduced iPS‐ECs, cells were fixed in 4% paraformaldehyde, permeabilized in 0.1% Triton X‐100 in PBS, and stained with selective primary antibodies against IL8RA, IL8RB, CCR2, or CCR5 and counterstained with DAPI as a nuclear marker 10. Stained ECs were mounted for fluorescent microscopic analysis with a computerized Leica DM6000 system 10.

### In Vivo Studies

#### Animals and Procedures

Twelve‐week‐old male Sprague Dawley Rats (Charles River Laboratories) were maintained at a constant temperature (24 ± 1°C), humidity (60 ± 5%), and light cycle (6 a.m. to 6 p.m.) and fed standard rat pellet diet ad libitum 10, 12, 22, 23. Protocols were approved by the Institutional Animal Care and Use Committee (IACUC) at the University of Alabama at Birmingham (UAB) and were consistent with the *Guide for the Care and Use of Laboratory Animals* published by the National Institutes of Health.

All rats were anesthetized with ketamine/xylazine (100/10 mg/kg BW) and subjected to implantation of a femoral venous catheter at 1 day before balloon injury of the right carotid artery (RCA). On the following day, rats were anesthetized with ketamine and xylazine and underwent balloon injury of the RCA as described previously 10, 12. Briefly, a 2F Fogarty balloon catheter (Baxter V. Mueller) was introduced through the external carotid artery and advanced into the thoracic aorta. The balloon was inflated with saline to distend the common carotid artery and was pulled back to the external carotid artery. After six repetitions of this procedure, the catheter was removed, the external carotid artery was ligated and the wound was closed. The left carotid artery (LCA) was not damaged and served as a control. The LCA was used as an uninjured control. Immediately after RCA injury, rat were randomly divided into five treatment groups and received i.v. transfusion of iPS‐ECs transduced with the empty adenoviral vector (RiPS‐AdNull‐iPS‐EC, 1.5 × 10^6^ cells per rat); iPS‐ECs overexpressing IL8RA/B (RiPS‐IL8RA/B‐ECs, 1.5 × 10^6^ cells per rat), iPS‐ECs overexpressing CCR2/5 (RiPS‐CCR2/5‐ECs, 1.5 × 10^6^ cells per rat), iPS‐ECs overexpressing both receptors (RiPS‐IL8RA/B+CCR2/5‐ECs, 1.5 × 10^6^ cells per rat), or saline (Vehicle control) 10, 12, 22, 23.

Rats were sacrificed 1 or 14 days after RCA injury for histological (neutrophils and monocytes/macrophages infiltration) and biochemical (inflammatory cytokine expression) or neointima formation analyses, respectively.

#### Real‐Time Quantitative RT‐PCR Analysis for mRNA Quantitation of Inflammatory Mediators

Rats were sacrificed 24 hours postinjury and RNA was extracted from the RCA and LCA, reverse transcribed to cDNA and amplified by real‐time quantitative PCR for the neutrophil attractant *CINC‐2β* (equivalent to IL8 in human) and the macrophage attractant *CCL2/MCP‐1*. The PCR primers for *CINC‐2β* were: forward 5′‐TCAGGGACTGTTGTGG‐3′ and reverse 3′‐TGACTTCTGTCTGGGTG‐5′: and for *CCL2/MCP‐1* were: forward 5′‐ATGCAGGTCTCTGTCACGCT‐3′ and reverse 3′‐GGTGCTGAAGTCCCTTAGGGT‐5′ 10, 12. Levels of specific mRNA were normalized using *18S RNA*, forward 5′‐GAAACGCTACCACATCC‐3′ and reverse 3′‐CACCAGACTTGCCCTCCA‐5′ 10, 12.

#### Measurement of Inflammatory Mediator Protein Levels

Protein levels of inflammatory cytokines were measured from protein homogenates of the injured RCA and uninjured LCA at 24 hours postinjury using a multiplexed rat‐specific magnetic beads‐based sandwich immunoassay kit (Milliplex Rat Cytokine/Chemokine Panel, Millipore, Billerica, MA) with the Luminex xMap analyzer as described previously 12, 22.

#### Immunohistochemical Assessment of Infiltration of Neutrophils and Monocytes/Macrophages into Injured Carotid Arteries

The avidin‐biotin‐peroxidase immunohistochemical technique was used to detect neutrophils (myeloperoxidase, MPO+) and monocytes/macrophages (ED1+) in paraffin‐embedded sections using specific primary antibodies and a VECTASTAIN ABC Kit (Vector Laboratories, Burlingame, CA) as described previously 10, 12, 22.

#### Morphometric Analysis of Neointima Formation

Neointimal and medial areas were determined using 5 μm cross‐sections of the injured RCA and uninjured LCA at 14 days postinjury, as described previously 10, 12. The computer‐based NIH ImageJ Morphometric system was used for morphometric analysis. Multiple H/E‐stained cross‐sections from the middle third of each vessel were used for quantitation, and the measurements were averaged for statistical analysis. The cross‐sectional areas of the vessel within the external elastic lamina (EEL area), within the internal elastic lamina (IEL area), and within the lumen (lumen area) were measured. Neointima formation in the injured RCA was determined by calculating the neointima‐to‐media area ratio 10, 12.

### Statistical Analysis

Age matched rats were use throughout. All statistical analyses were used the SigmaStat statistical package (Version 3.5). A student *t* test was used for comparisons between individual groups and one‐way ANOVA was used for comparisons across multiple groups. When the *F* test ANOVA was statistically significant, a Tukey test was applied for further analysis 10, 12. Results are presented as means ± SEM. All statistically significant differences are reported when *p* < .05.

## Results

### RiPS‐ECs Express EC Markers

**Figure 1:**
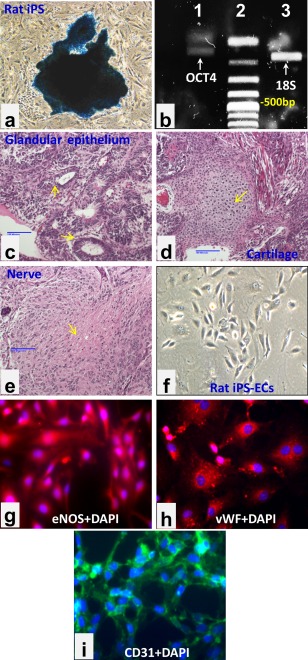
Rat‐induced pluripotent stem (RiPS) cells were generated from rat embryonic fibroblasts and then differentiated to endothelial cells (RiPS‐ECs). **(A)**: RiPS Cells were stained with alkaline phosphatase (a stem cell marker); **(B)** reverse transcription‐PCR products of transduced transcriptional factor OCT4 (144 base pair [bp]) and 18S (168 bp, as a PCR control) in RiPS cells; **(C–E)** teratoma containing tissue derived from all three germ layers was obtained when RiPS cells were injected into the dorsal flanks of severe combined immunodeficiency mice. Photo micrograph: magnification ×100 (Scale bars = 100 µm). **(C)**: Glandular epithelium endoderm derived from endoderm; **(D)** cartilage derived from mesoderm; **(E)** nerve tissue derived from ectoderm; **(F–I)** RiPS‐differentiated ECs exhibited typical cobblestone morphology (F) and expressed eNOS (G), vWF (H), and CD31 (I). Photo micrograph: magnification x200 (F), magnification x400 (G–I). Abbreviations: DAPI, 4′,6‐diamidino‐2‐phenylindole; eNOS, endothelial nitric oxide synthase; iPS, induced pluripotent stem; vWF, von Willebrand factor.

RiPS cells generated from rat embryonic fibroblasts expressed alkaline phosphatase, a stem cell marker (Fig. 1A). Oct4 mRNA expression was detected in RiPS cells (Fig. 1B). When these iPS cells were injected into the dorsal flanks of SCID mice, teratoma containing tissue derived from all three germ layers was obtained (Fig. 1C–1E). These results demonstrate that the rat embryonic fibroblasts have been effectively reprogrammed to iPS cells. Differentiated RiPS‐ECs showed a monolayer cobblestone EC phenotype and expressed the EC markers eNOS, vWF, and CD31 (Fig. 1F–1I). Oct4 mRNA was not detected in differentiated RiPS‐ECs (data not shown).

### Adenoviral Transduced RiPS‐ECs Overexpress IL8RA/RB and CCR2/5 Receptors

**Figure 2:**
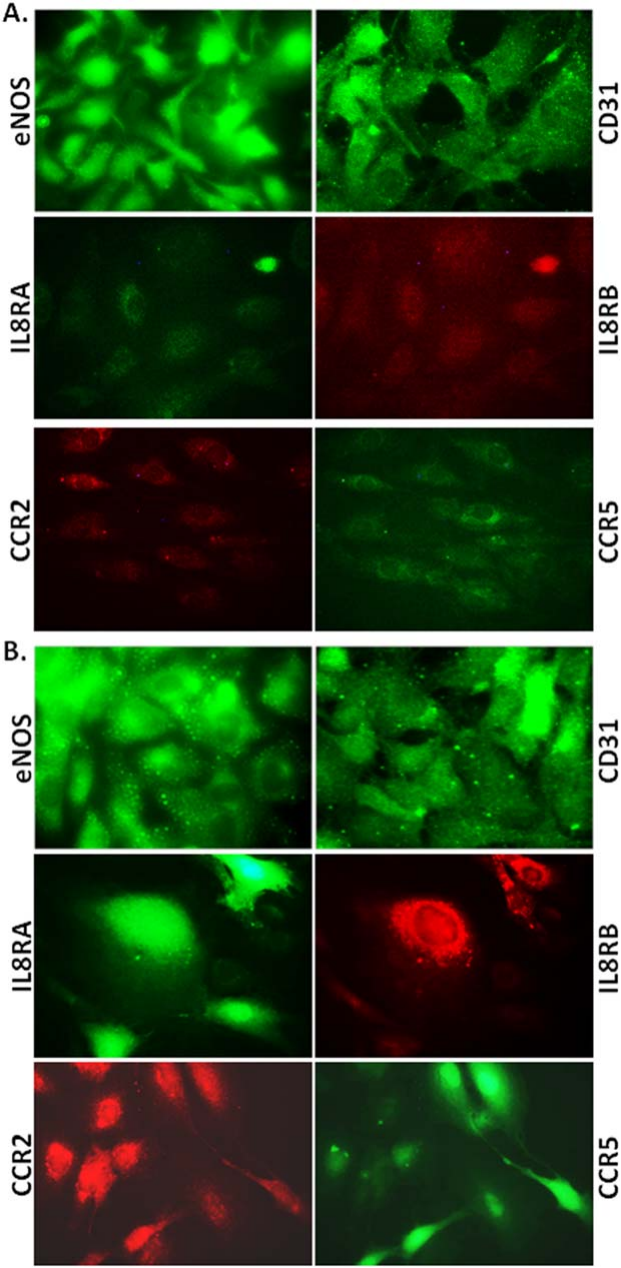
Characterization of RiPS‐ECs transduced with IL8RA/B, CCR2/5, or AdNull adenoviral vectors. **(A)**: Fluorescence micrographs showing expression of endothelial cell (EC) markers endothelial nitric oxide synthase (eNOS) and CD31, and very low level of expression of IL8RA/B and CCR2/5 in RiPS‐ECs transduced with empty adenovirus carrying only green fluorescent protein gene. **(B)**: Flurorescence micrographs showing overexpression of IL8RA/B and CCR2/5 in iPS‐ECs transduced with Ad‐CXCR1/2 and Ad‐CCR2/5. The adenoviral transduction did not alter the expression of eNOS or CD31 in these cells. Photo micrograph: magnification ×400. Abbreviations: CCR, C‐C chemokine receptors; IL, interleukin; RiPS, rat‐induced pluripotent stem cells.

Transduction with adenoviral vectors carrying IL8RA, IL8RB, CCR2, and/or CCR2/5 genes caused overexpression of these receptors in RiPS‐ECs 72 hours after transduction (Fig. 2A, 2B). The adenoviral transduction did not alter the expression of eNOS or CD31 in RiPS‐ECs (Fig. 2A, 2B).

### Transfusion of RiPS‐ECs Overexpressing IL8RA/RB and/or CCR2/5 Decreases Expression of Neutrophil and Macrophage Chemoattractants in Injured RCA

**Figure 3:**
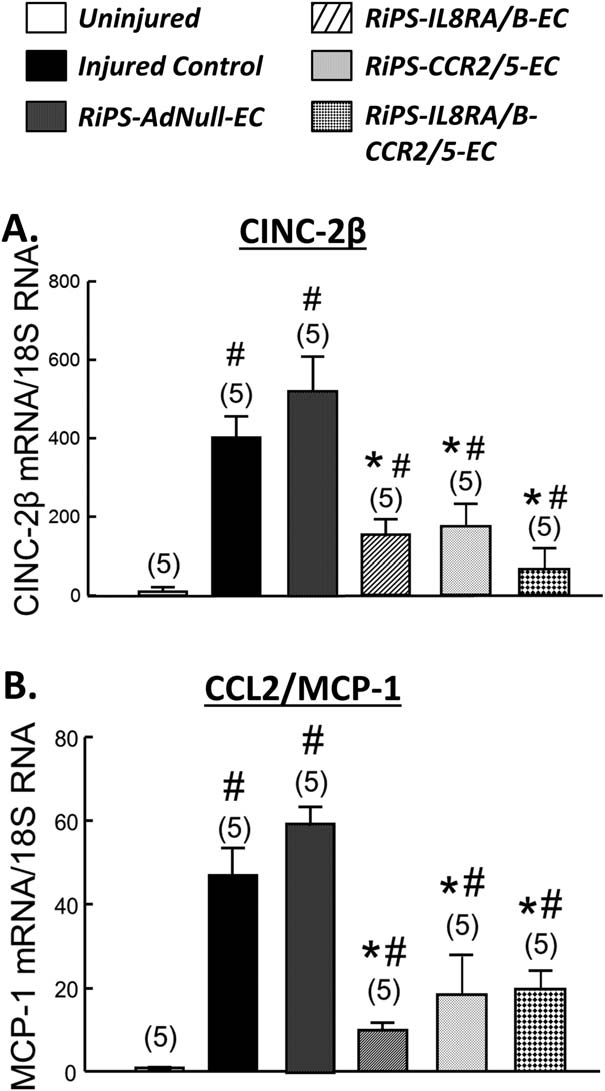
Effects of RiPS‐AdNull‐ECs, RiPS‐IL8RA/B‐ECs and/or RiPS‐CCR2/5‐ECs transfusion on mRNA of CINC‐2β **(A)** and CCL2/MCP‐1 **(B)** in injured right carotid arteries (RCA) at 24 hours postinjury and cell transfusion. mRNA levels were normalized using 18S RNA to correct for differences in total RNA loading. Results are means ± SEM, (*n*) = number of vessels. # *p* < .05, compared to Uninjured control group; * *p* < .05, compared to Injured control group. Abbreviations: CCL, C‐C chemokine ligand; CCR, C‐C chemokine receptors; CINC, cytokine‐induced neutrophil chemoattractant; EC, endothelial cell; IL, interleukin; MCP, monocyte chemoattractant protein; RiPS, rat‐induced pluripotent stem cells.

Balloon injury significantly increased mRNA expression of the neutrophil chemoattractant CINC‐2β (the rat homolog of IL8) and the macrophage chemoattractant CCL2/MCP‐1 in RCA at 24 hours postinjury. Transfusion of RiPS‐ECs overexpressing IL8RA/RB (RiPS‐IL8RA/B‐ECs), CCR2/5 (RiPS‐CCR2/5‐ECs), or both (RiPS‐IL8RA/B‐CCR2/5‐ECs) significantly decreased (>65%) the injury‐induced CINC‐2β and CCL2/MCP‐1 mRNA expression in RCA. Transfusion of RiPS‐AdNull‐ECs did not alter mRNA levels of Cinc‐2β or CCL2/MCP‐1 in RCA (Fig. 3).

**Figure 4:**
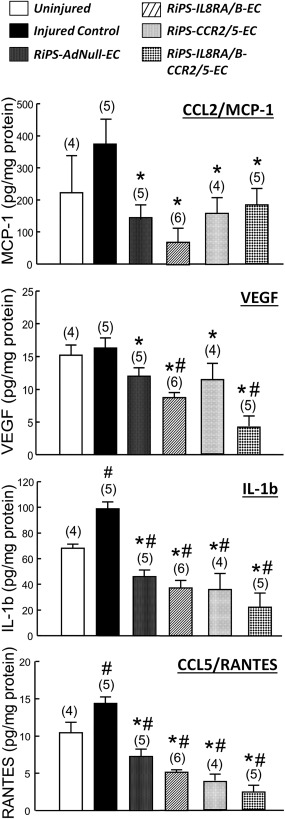
Effects of RiPS‐AdNull‐ECs, RiPS‐IL8RA/B‐ECs, and/or RiPS‐CCR2/5‐ECs transfusion on protein levels of chemokines and cytokines in injured right carotid artery at 24 hours postinjury and cell transfusion. Cytokine/chemokine levels were measured in whole vessel homogenates using commercially available Multiplexed rat specific magnetic beads‐based sandwich immunoassay kits with a Luminex xMAP analyzer (Milliplex Rat Cytokine/Chemokine Panel, Millipore). Results are means ± SEM, (*n*) = number of vessels. # *p* < .05, compared to Uninjured control group; * *p* < .05, compared to Injured control group. **(A)**: CCL2/MCP‐1, **(B)** vascular endothelial growth factor, **(C)** IL‐1β (interleukin 1‐β), and **(D)** CCL5/RANTES (regulated on activation, normal T cell expressed and secreted). Abbreviations: CCL, C‐C chemokine ligand; CCR, C‐C chemokine receptors; EC, endothelial cell; IL, interleukin; MCP, monocyte chemoattractant protein; RANTES, regulated on activation, normal T cell expressed and secreted; RiPS, rat‐induced pluripotent stem cells; VEGF, vascular endothelial growth factor.

Similarly, balloon injury caused increases in the protein levels of the inflammatory cytokines CCL2/MCP‐1, vascular endothelial growth factor, interleukin (IL)−1b, and CCL5/regulated on activation, normal T cell expressed and secreted (RANTES) in the injured RCA, compared to the uninjured LCA, at 24 hours postinjury. Levels of these inflammatory cytokines were reduced in injured RCA of rats transfused with RiPS‐IL8RA/B‐ECs, RiPS‐CCR2/5‐ECs, or RiPS‐IL8RA/B‐CCR2/5‐ECs, as well as in RCA of rats transfused with RiPS‐AdNull‐ECs (Fig. 4).

### RiPS‐ECs Overexpressing IL8RA/RB or CCR2/5 Decrease Migration of Neutrophils and Monocytes/Macrophages into Injured RCA

**Figure 5:**
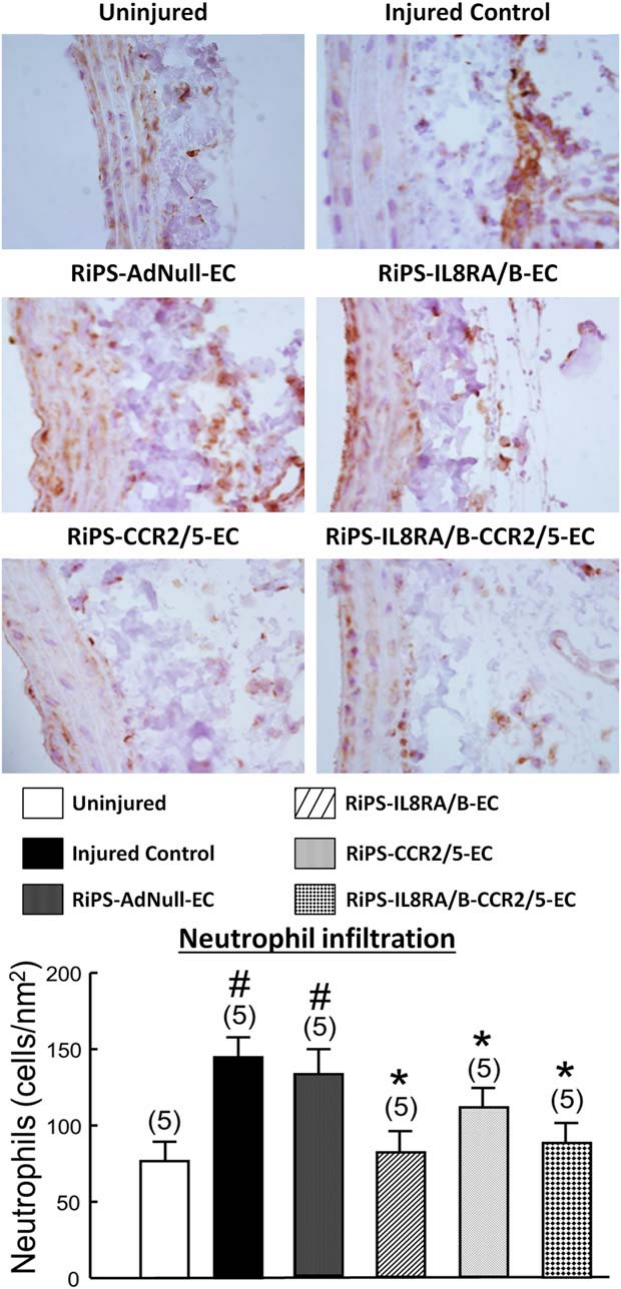
Effects of RiPS‐AdNull‐ECs, RiPS‐IL8RA/B‐ECs, and/or RiPS‐CCR2/5‐ECs transfusion on neutrophils infiltration into injured RCA at 24 hours postinjury and cell transfusion. **Top**: Representative photo micrographs of neutrophil (MPO+) infiltration into adventitia of rat arteries. Photo micrograph: magnification ×630. **Bottom**: Numbers of MPO+ cells were counted in 6 fields of cross‐sections of each vessel. Results are means ± SEM, (*n*) = number of vessels. # *p* < .05, compared to Uninjured control group; * *p* < .05, compared to Injured control group. Abbreviations: CCR, C‐C chemokine receptors; EC, endothelial cell; MPO, myeloperoxidase; IL, interleukin; RiPS, rat‐induced pluripotent stem cells.

**Figure 6:**
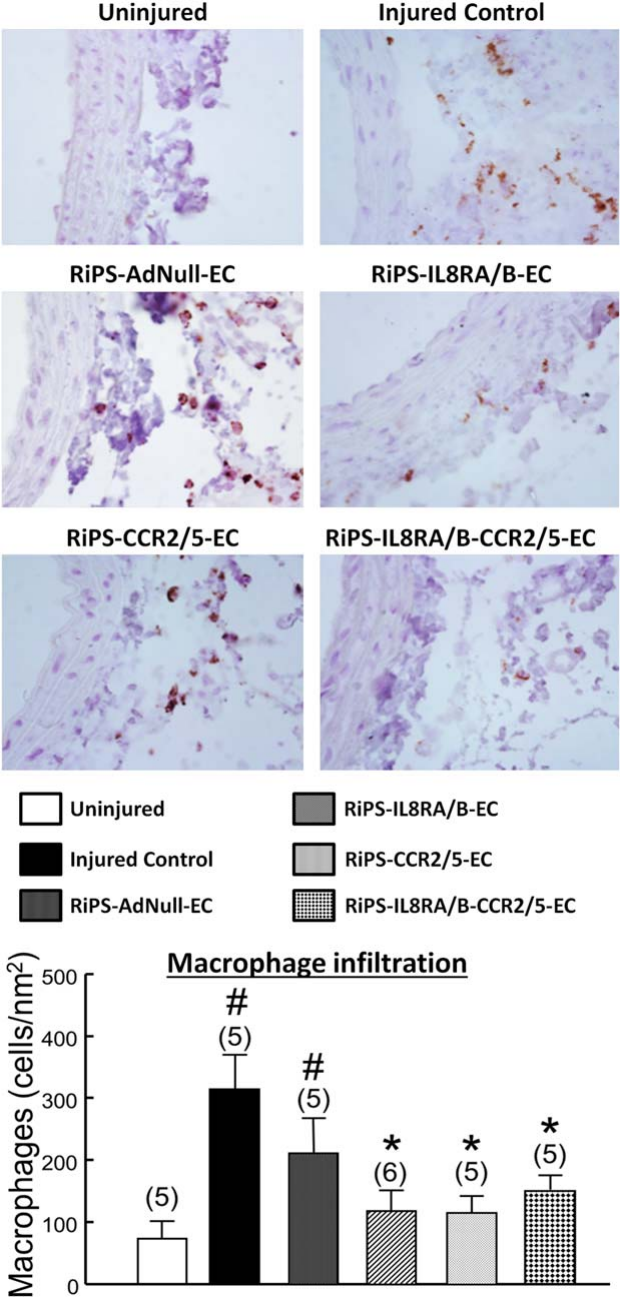
Effects of RiPS‐AdNull‐ECs, RiPS‐IL8RA/B‐ECs, and/or RiPS‐CCR2/5‐ECs transfusion on monocytes/macrophages infiltration into injured RCA at 24 hours postinjury and cell transfusion. **Top**: Representative micrographs of monocytes/macrophages (ED1+) infiltration into adventitia of rat arteries. Photo micrograph: magnification ×630. **Bottom**: Numbers of ED1+ cells were counted in 6 fields of cross‐sections of each vessel. Results are means ± SEM, (*n*) = number of vessels. # *p* < .05, compared to Uninjured control group; * *p* < .05, compared to Injured control group. Abbreviations: CCR, C‐C chemokine receptors; EC, endothelial cell; IL, interleukin; RiPS, rat‐induced pluripotent stem cells.

Balloon injury significantly stimulated infiltration of neutrophils and monocytes/macrophages into adventitia of injured RCA at 24 hours postinjury. Transfusion of RiPS‐IL8RA/B‐ECs, RiPS‐CCR2/5‐ECs, or RiPS‐IL8RA/B‐CCR2/5‐ECs significantly decreased (>65%) the injury‐induced neutrophil and monocyte/macrophage infiltration, but transfusion with RiPS‐AdNull‐ECs did not alter inflammatory leukocyte infiltration into RCA (Figs. 5 and 6).

### Transfusion of RiPS‐RA/RB‐ECs, RiPS‐CCR2/5‐ECs, or RiPS‐RA/RB‐CCR2/5‐ECs Inhibits Neointima Formation in Injured RCA

**Figure 7:**
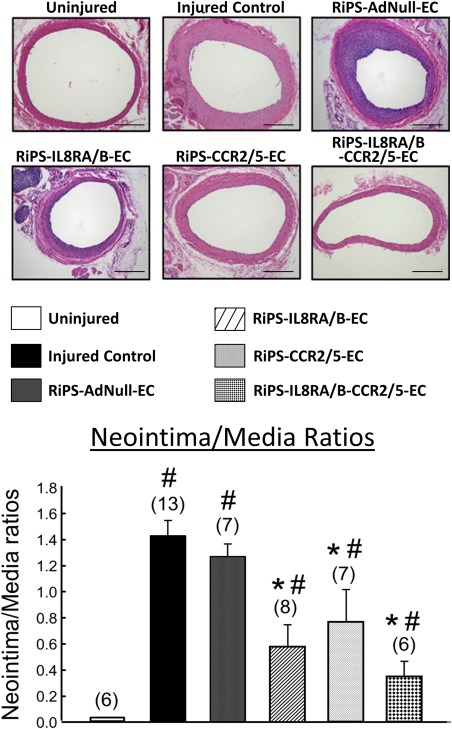
Effects of RiPS‐AdNull‐ECs, RiPS‐IL8RA/B‐ECs, and/or RiPS‐CCR2/5‐ECs transfusion on neointima formation 14 days post carotid arterial injury and cell transfusion. **Top**: Representative H/E‐stained cross‐sections of injured RCA and un‐injured left carotid artery at 14 days post balloon injury. Photo micrograph: magnification ×100 (Scale bars = 200 µm). **Bottom**: Quantification of neointima to media ratios. The media areas were not different among the six groups. Results are means ± SEM, (*n*) = number of vessels. # *p* < .05, compared to Uninjured control group; * *p* < .05, compared to Injured control group. Abbreviations: CCR, C‐C chemokine receptors; EC, endothelial cell; IL, interleukin; RiPS, rat‐induced pluripotent stem cells.

Balloon injury‐induced neointima formation in RCA was inhibited by transfusion with RiPS‐IL8RA/RB‐ECs, RiPS‐CCR2/5‐ECs, or RiPS‐IL8RA/RB‐CCR2/5‐ECs, but not control RiPS‐AdNull‐ECs. RiPS‐IL8RA/RB‐ECs and RiPS‐CCR2/5‐ECs were equally effective in preventing neointima formation. The combination of IL8RA/B and CCR2/5 overexpression in RiPS‐ECs had a slight, statistically nonsignificant, additive inhibitory effect on neointima formation in RCA (Fig. 7).

## Discussion

This study demonstrated that targeted cell therapy using ECs derived from RiPS cells overexpressing IL8RA/RB and/or CCR2/5 attenuates inflammation and neointima formation after balloon injury of the carotid artery. We were able to successfully generate rat iPS cells from rat embryonic fibroblasts and orient them into ECs. Intravenous infusion of RiPS‐ECs overexpressing IL8RA/B and/or CCR2/5 outcompeted inhibited neutrophils and macrophages in the injured carotid artery, decreased inflammatory cytokine expression at 24‐hour postinjury, and reduced neointima formation at 2 weeks postinjury. Thus, we have shown that inhibiting neutrophil and/or macrophage infiltration during the early inflammatory response to endoluminal vascular injury decreases subsequent adverse vascular remodeling.

Use of iPS cells can eventually enable the development of autologous cell therapy and thus overcome the major hurdle that stands in the way of practical application of cell therapy for human diseases. The successful use of rat iPS derived into ECs in preventing the inflammation and adverse vascular remodeling that results from acute vascular injury provides proof of concept in support of potential therapeutic interventions using autologous cell therapy, as described by Volz et al. 24. A major concern of using cell therapy as a potential treatment for human diseases is rejection of the transplanted cells; autologous cell therapy overcomes the obstacle of rejection 24.

We have previously demonstrated that targeted cell therapy with RAECs and human iPS‐ECs overexpressing IL8RA/RB inhibits early inflammatory mediator expression, inflammatory cell infiltration, and subsequent neointima formation in a variety of models of acute vascular injury, including balloon‐injured rat carotid arteries 10, 12, monocrotalin‐induced acute lung injury 22, and coronary ligation‐induced myocardial infarction 23. In the current study, RiPS‐ECs overexpressing IL8RA/RB achieved similar protective effects against inflammatory responses and neointima formation, indicating consistency of targeting neutrophil chemotaxis. Our previous work has also shown that inhibiting both IL8RA and RB concurrently is more effective at reducing neointima formation than inhibiting either receptor alone 10. Further, we have shown that our targeted cell therapy is EC specific, since smooth muscle cells overexpressing IL8RA/B did not inhibit neointima formation in the rat carotid injury model 10. In the current study, there were decreases in inflammatory cytokine expression and a trend toward decreases in neutrophil and monocyte/macrophage infiltration in injured arteries of RiPS‐AdNull‐ECs transfused animals. We postulate that these anti‐inflammatory effects may be related to low levels of IL8RA/B and CCR2/5 expression in RiPS‐AdNull‐ECs. However, the attenuation of the early inflammatory response in the injured RCA of rats treated with RiPS‐Null‐ECs was not reflected in a reduction in neointima formation, consistent with our previous findings in balloon‐injured carotid arteries of rats treated with human iPS‐AdNull‐ECs versus human iPS‐ILR8A/B‐ECs 12. As we previously demonstrated, both decreasing proinflammatory responses and accelerating reendothelialization contribute to inhibition of neointima formation in balloon‐injured rat carotid arteries, and transfusion of IL8 receptor‐targeted ECs is more effective than transfusion of equal numbers of untargeted ECs in inhibiting the later phases of the inflammatory response, for example, cell migration, proliferation, remodeling of matrix proteins, and neointima formation, as shown in our previous studies 12.

A novel aspect of the current study is the demonstration that RiPS‐ECs overexpressing CCR2/5 protect the vasculature against endoluminal injury. The chemokine receptor CCR2 is expressed abundantly on the “inflammatory” subset of blood monocytes 25, as well as other immune/inflammatory cell types such as dendritic cells and memory Th1 cells 26, 27. CCR2 is a G protein‐coupled receptor that binds multiple ligands, characterized as macrophage chemoattractant proteins, including CCL2/MCP‐1, CCL8/MCP‐2, CCL7/MCP‐3, and CCL13/MCP‐4. Of these ligands, MCP‐1 is the best studied, and CCR2 is considered to be its major receptor 13, 28, 29. The CCR2/MCP‐1 axis mediates emigration of inflammatory monocytes from bone marrow to blood and immigration of blood monocytes from blood to inflamed tissues 14, 30, 31, 32, 33, 34, 35, 36.

CCR5 is expressed predominantly on macrophages that have been differentiated from blood monocytes and Th1 cells that have been activated in response to inflammatory stimuli 13, 37, and is also expressed on nonimmune cells such as osteoclasts 38 and VSMCs 39. CCR5 is a G protein‐coupled receptor that binds multiple ligands, including CCL4/macrophage inflammatory protein‐1β (MIP‐1β), CCL5/RANTES, CCL3/MIP‐1α, CCL8/MCP‐2, and CCL3‐like 1 (CCL3L1)/LD78. CCR5 contributes to the survival of macrophages during inflammation and infection 40 and may function to retain tissue macrophages in inflamed tissue 41. Consistent with their key role in monocyte trafficking and macrophage survival/retention, CCR2/5 have been shown to drive inflammation in a number of animal models of vascular disease, including atherosclerosis 42, 43, 44, 45, 46, 47, 48, 49, 50, 51, and restenosis 52, 53, 54, 55, 56, 57, 58, 59, 60, 61 following revascularization. Genetic ablation 52, 59 or pharmacologic inhibition of CCR2/5 [54–57, 61] has been shown to reduce macrophage infiltration and neointima formation following vascular injury. However, systemic deletion of CCR2/5 gene expression 62, 63, 64, 65, 66 in mice and blocking of CCR5 with a pharmacologic antagonist in humans 67 have been linked to hepatotoxicity. Accordingly, this approach likely has little translational potential for treatment of CVD. Our strategy utilizing dual targeting of CCR2 and CCR5 by RiPS‐CCR2/5‐ECs appears to be a more efficacious and safer approach with greater potential for translation to treatment of patient with CVD.

Interestingly, inhibiting both neutrophils and macrophages with RiPS‐IL8RA/B‐CCR2/5‐ECs, did not have a cumulative effect on injury‐induced vascular inflammatory responses or neointima formation. Further research is needed to delineate the detailed cellular/molecular mechanisms and signaling pathways by which these leukocyte subtypes mediate the vascular injury response. However, our data clearly indicate that targeting delivery of RiPS‐ECs overexpressing either IL8RA/B or CCR2/5 is an effective treatment for acute vascular injury.

## Conclusion

Transfused RiPS‐ECs overexpressing IL8RA/B and/or CCR2/5 migrate into injured vessels and compete with neutrophil/macrophage infiltration, preventing inflammatory responses and neointima formation. Targeted delivery of iPS‐ECs with a homing device to inflammatory mediators in injured arteries provides a novel strategy for the treatment of CVDs.

## Author Contributions

S.G.: Conception and experimental design, data collection, analysis and interpretation, manuscript writing; X.Z.: Conception and experimental design, data collection and analysis; Y.‐F.C.: Financial support, conception and experimental design, data analysis and interpretation, manuscript writing, final approval of manuscript; S.H.L.: Data collection, analysis and interpretation; F.G.H.: Data analysis and interpretation; T.M.T., C.W.S., L.‐C.W.: Generation of experimental material; S.O.: Conception and experimental design, data interpretation, manuscript writing, final approval of manuscript; D.X.: Conception and experimental design, data collection, analysis and interpretation, manuscript writing, final approval of manuscript

## Disclosures of Potential Conflicts of Interest

The authors indicate no potential conflicts of interest.
